# Use of Agave Bagasse and *Lactococcus lactis* in Sourdough Production: Drying Effects on Bioactive Compounds

**DOI:** 10.3390/foods14101748

**Published:** 2025-05-14

**Authors:** Paola Itzel Bautista-Espinoza, Aniello Falciano, Rosalía Reynoso-Camacho, Everardo Mares-Mares, Silvia Lorena Amaya-Llamo, Carlos Regalado-González, Prospero Di Pierro

**Affiliations:** 1 Departamento de Investigación y Posgrado en Alimentos, Facultad de Química, Universidad Autónoma de Querétaro, Cerro de las Campanas s/n, Querétaro 76010, Mexico; paolaespinoza9606@gmail.com (P.I.B.-E.); rrcamachomx@yahoo.com.mx (R.R.-C.); samayal@uaq.mx (S.L.A.-L.); regcarlos@gmail.com (C.R.-G.); 2 Centre for Food Innovation and Development in the Food Industry, University of Naples Federico II, 80055 Naples, Italy; prospero.dipierro@unina.it; 3 Department of Agricultural Sciences, University of Naples Federico II, 80055 Naples, Italy; 4 ITS de Guanajuato (ITESG), Tecnologico Nacional de México, Carretera Guanajuato-Puentecillas Km 10.5, Puentecillas, Guanajuato 36262, Mexico; emares@itesg.edu.mx

**Keywords:** agave, functional food, lactic acid bacteria, sustainable ingredients, drying

## Abstract

The wastage of by-products generated in the food industry is an issue that should be addressed by determining a second use for these products, with sourdough fermentation being the most popular technology used. The aim of this research was to evaluate the impact of adding agave bagasse (AB) and *Lactococcus lactis* NRRL B-50307 to sourdough that was later used in the formulation of bread rolls. Five treatments were tested: B1: wheat flour; BI2: wheat flour inoculated with *L. lactis* (1 × 10^6^ CFU/mL); C10: wheat flour + AB (10% *w*/*w*); T5: 5% AB + wheat flour inoculated with *L. lactis* (1 × 10^6^ CFU/mL); and T10: 10% AB + wheat flour inoculated with *L. lactis* (1 × 10^6^ CFU/mL). Sourdoughs were back-slopped daily for 6 days, dried in a climatic chamber, reactivated, and left to ferment for 24 h. Samples of each treatment of dried and reactivated sourdough were collected and tests for antioxidant activity (DPPH and ABTS), total amino acid content (OPA), and phenolic and flavonoid content were performed. Phenolic compounds and flavonoids decreased when the sourdough was dried (1.5 to 2.0 mg/g of quercetin); however, an increase in bioactive compounds was observed after reactivation, with the treatments with AB recording the highest values (2.5 mg/g). The DPPH and ABTS tests showed that T10 had the highest activity (25% and 23%, respectively). The OPA results showed an increment in amino acid content (2.0 mg lysine/g), indicating proteolysis. The fermentation curves showed that leavening time was achieved after 600 min of fermentation. AB addition did not affect the viscosity of the sourdough rolls. Sourdough with added AB and *L. lactis* provided a novel approach to achieve more sustainable baked goods. The drying process decreased the sourdough’s bioactive compounds, which were recovered after reactivation.

## 1. Introduction

Currently, sourdough bread is one of the most popular bakery products worldwide, thanks to consumers seeking this product due to its multiple sensory and nutritional properties. The fermentation of sourdough is one of the most complex processes in the food industry due to the interaction of the metabolism of both lactic acid bacteria (LAB) and yeasts [1]. On the other hand, it is known that sourdough fermentation can improve the availability of bioactive compounds such as phenols and flavonoids [2] due to various mechanisms such as nutrient competition and utilization, acidity regulation, flavor formation, etc. [3].

Grains, such as wheat, contain a high concentration of phytochemicals, including phenolic compounds (both flavonoid and non-flavonoid), phenolic acids, and phytates, primarily localized in the outer layers of the grain [4]. However, the bioavailability of these compounds is significantly affected by processing methods, particularly milling, which removes the bran and germ, leading to substantial phytochemical losses. Additionally, baking induces further modifications in bioactive compounds due to thermal degradation, oxidation, and Maillard reactions, which can either decrease or, in some cases, transform and enhance the functionality of certain compounds.

To counteract these losses, the bakery industry has been exploring various biotechnological approaches, such as fermentation, to improve the bioavailability and stability of bioactive compounds derived from plant sources. Fermentation, mediated by selected microbial strains, has been shown to enhance the content of phenolic compounds through the enzymatic hydrolysis of bound phenolics, while also promoting the synthesis of antimicrobial, anti-inflammatory, and flavor-enhancing metabolites [5].

Furthermore, cereal and vegetable by-products represent a major sustainability challenge for the food industry. These agro-industrial residues are rich in bioactive compounds, polysaccharides, and dietary fiber, which could be repurposed to improve the nutritional quality and functionality of food products. Their incorporation into bakery formulations not only contributes to the development of healthier foods but also mitigates the environmental impact of food waste [6]

In Mexico, agave bagasse (AB) is one of the most abundant industrial by-products, primarily generated during the production of tequila and mezcal. This lignocellulosic residue represents approximately 40% of the whole agave plant [7] and is typically discarded, despite its valuable composition. AB is mainly composed of cellulose (≈43%), hemicellulose (≈19%), and lignin (≈15%), making it a rich source of dietary fiber [8]. Its average moisture content ranges between 60% and 75%, which limits its stability and shelf life, and contributes to its underutilization.

Although AB has been studied for applications such as biofuel production and animal feed, its potential as a food ingredient remains largely unexplored. In particular, its bound phenolic compounds content and antioxidant potential is underreported. Fermentation processes, especially sourdough fermentation, could enhance the release of these bioactive compounds, improving the nutritional and functional value of AB. Moreover, the addition of selected lactic acid bacteria (LAB), such as *Lactococcus lactis*, may promote this release through acidification and enzymatic activity [9].

One of the main challenges in sourdough production is its limited portability due to its high moisture content, volume, and need for frequent refreshments. Drying has been proposed as a practical method to improve sourdough storage and transportation. However, the effect of drying on the stability of bioactive compounds is not fully understood and requires further investigation.

Therefore, the aim of this study was to evaluate the effect of incorporating agave bagasse and *Lactococcus lactis* NRRL B-50307 into mature sourdough, with a focus on the content of bioactive compounds before and after drying. Additionally, the study assessed the performance of the reactivated sourdough during bread roll preparation and leavening, in order to explore its potential as a sustainable functional ingredient.

## 2. Materials and Methods

### 2.1. Chemicals

The following chemicals were utilized: 1,1-diphenyl-2-picrylhydrazyl (DPPH); 2,2′-azino-bis (3-ethylbenzothiazoline-6-sulfonic acid) (ABTS); NaCl; K_2_HPO_4_; EDTA (Sigma-Aldrich, St. Louis, MO, USA); absolute methanol; o-phthalaldehyde detection kit; lactic, acetic, and citric determination kits (Megazyme, Wicklow, Ireland); potato dextrose agar (PDA); total mesophilic bacteria agar (PCA); yeast agar (YPD); MRS and M17 broth (Bioxon, Kowale, Polonia).

### 2.2. Organic Material

Wheat flour 00 was purchased online from Caputo company (Antimo Caputo S.r.l., Napoli, Italy). The flour contained 12.5 g protein, 0.006 g salt, 72 g carbohydrates, 3 g fiber, and 1 g lipids. Agave bagasse (AB) was produced by a local distillery in Penjamo city, Mexico. Tap water, salt, and olive oil were purchased from local markets. *Lactococcus lactis* strains (NRRL B-50307) were obtained from a private collection at Autonomous University of Querétaro (Querétaro, Mexico).

### 2.3. Methods

#### 2.3.1. Sourdough Formulation

Previously, different AB concentrations have been tested to determine the best sourdough formulation [10]. Two treatments were chosen for displaying the best technological conditions: T5 (wheat sourdough with 5% of agave bagasse added) and T10 (wheat sourdough with 10% of agave bagasse added). Both treatments were inoculated with 1 × 10^6^ CFU/mL *L. lactis*. Three control treatments were established: B1 (wheat sourdough), BI2 (wheat sourdough inoculated with *L. lactis*), and C10 (agave bagasse with wheat flour). All treatments were refreshed daily (by adding water, wheat flour, AB, and the *L. lactis* strain while removing a part of the sourdough) until day 6 of maturation at room temperature (25 °C ± 2 °C).

#### 2.3.2. Drying and Reactivation Process of the Sourdough

The sourdoughs were dried after they reached the maturation stage in a sterile climatic chamber (KK 115, Pol-Eko, Wodzisław Śląsk, Poland). The liquid sourdoughs were poured onto rectangular trays and extended with a spatula until a thin layer (approximately 1 cm thick) was obtained. The drying conditions were set as follows: 35 °C ± 2 °C, 70% humidity, and 25% fan speed. Once the sourdoughs were completely dried (humidity percentage of 7.34 ± 0.2%), they were pulverized (Nutribullet NBR-0504, Ciudad de México, Mexico) and sieved to obtain a uniform particle size (200 µm). The flours were then vacuum packaged until use (Eco vac 400D TB, ISG Pack, Roma, Italy).

To measure the impact of the drying process on the sourdough, the microbial counts of the different formulations were evaluated under different conditions: fresh mature sourdough (FS), dried sourdough (DS), dried sourdough re-activated (RS1) with no fermentation, and re-activated sourdough after 24 h of fermentation (RS2). Reactivation and fermentation processes were performed by following the refreshment and fermentation conditions mentioned in previous sections.

#### 2.3.3. Microbial Viability of the Sourdoughs

Each sample (1 g) was weighed and diluted in 9 mL of sterile water and mixed in a stomacher (Stomacher 400, Seward, UK). Solutions were inoculated in MRS agar for lactic acid bacteria, YPD agar for yeasts, PCA for total aerobic mesophilic bacteria, and PDA for fungus following the manufacturer’s instructions for each medium. Petri dishes were incubated and microbial counts expressed as log CFU/g of sourdough.

#### 2.3.4. Preparation of Methanolic Extracts for Analysis

Each dry sample (1 g) was mixed with 25 mL of 70% (*v*/*v*) aqueous methanol and gently swirled at room temperature for 2 h. The mixture was then centrifuged at 12,000× *g* for 15 min at 20 °C. The resulting supernatants were collected and kept on ice in darkness, while the remaining pellets underwent a second extraction. Finally, all supernatants were combined and stored at −23 °C until further analysis.

#### 2.3.5. Total Phenolic (TPC) and Flavonoid (TFC) Compounds Determination

The total phenolic (TPC) and flavonoid compounds were evaluated following the method described by Falciano et al. [11]. For TPC, 50 μL of the extracts were combined with 70 μL of Folin–Ciocalteu (FC) reagent and 880 μL of distilled water. This mixture was vigorously vortexed for 1 min and left to incubate at room temperature for 5 min. Afterwards, 530 μL of distilled water and 70 μL of 7.5% (*w*/*v*) sodium carbonate were added to the tubes. The samples were then incubated in darkness at 25 °C for 15 min, and the absorbance was measured at 760 nm using a UV–VIS spectrophotometer (V-730, JASCO International Co., Ltd., Sennincho Hachioji, Japan) and the TPC values were expressed as mg of Gallic Acid Equivalent (GAE)/g (dw).

For TFC, 0.5 mL of the extracts were mixed with 1.5 mL of 80% methanol. The mixture was then combined with 0.1 mL of 1 M potassium acetate, 0.1 mL of 10% aluminum chloride, and 2.8 mL of distilled water. After thorough mixing, the solution was incubated at room temperature for 30 min. The absorbance was then measured at 410 nm (using the same spectrophotometer) and the results were reported as mg of Quercetin Equivalent (QE)/g (dw).

#### 2.3.6. Antioxidant Activity by DPPH and ABTS Methods

For the determination of antioxidant activity, a DPPH (2,2-diphenyl-1-picrylhydrazyl) assay and ABTS (2,2′-azino-bis-(3-ethylbenzothiazoline-6-sulfonic) acid) assay were performed. For the DPPH method, 950 µL of DPPH reagent were mixed with 50 µL of sample and left in darkness for 1 h at room temperature. Absorbances were measured at 517 nm, with the blank being DPPH reagent with methanol. For the ABTS determination, 1 mL of ABTS solution 7 mM was added to 50 µL of sample and 50 µL of absolute methanol. The sample was mixed and then incubated for 5 min in darkness. The blank was ABTS reagent with ethanol. Absorbance was read at 732 nm (using the spectrophotometer reported in the previous paragraph). To calculate the percentage of antioxidant activity in both methods, the following Equation (1) was used:

(1) $$\%\;antioxidant\;activity=\frac{\left(Ab-Abs\right)}{Ab}\times 100\;$$

where

Ab = absorbance of the control;

Abs = absorbance of the sample.

#### 2.3.7. Free Amino Acid Content (OPA) Determination

The determination of free amino acid content was carried out using the o-phthalaldehyde (OPA) method: 0.05 g of sample was weighed and mixed with 1 mL of trichloroacetic acid (TCA) 0.75 M. The mix was homogenized and stored at 4 °C for 30 min. Afterward, samples were centrifuged (4000× *g*) for 10 min and 100 μL of the supernatant was mixed with 100 μL of OPA reagent (ThermoFisher Scientific, Waltham, MA, USA). The sample was left to react for 5 min and absorbance was measured at 340 nm, using lysine for the calibration curve.

#### 2.3.8. Dough Preparation: Study of Leavening and Evaluation of Consistency

To measure the impact of the different sourdough formulations, doughs were formed as follows: 300 g of re-activated sourdough was mixed with 1000 g of whole wheat flour, 50 g of olive oil, 1 g of salt, and 600 mL of tap water. The ingredients were mixed using a professional mixer and 250 g dough balls were formed from the final mass, which were then placed for 24 h in a climatic chamber (20 ± 2 °C and 80% relative humidity).

Image analysis was used to evaluate the volume of the dough during the leavening phase [12]. A camera (GoPro Hero 13, RageCams, Sparta, MI, USA) was placed inside the incubation chamber and programmed to take photographs every 30 min, automatically. These images were then analyzed using the free, open-source image processing software ImageJ (Java2HTML v. 1.5, National Institutes of Health, Bethesda, MD, USA) in order to measure the volume using the Pappus–Guldinus theorem, according to the following Equation (2):

(2) V = 2πAM

where A is the area of the cross-section and M is the distance from the centroid of the section to the axis of rotation. The results were expressed as the ratio of V_1_/V_0_. V_1_: volume at *n*° time, mL; V_0_: volume at 0th time, mL.

The consistency was evaluated when the dough reached three-quarters of the maximum volume [13] and was measured following the protocol by Gys et al. [14] using a Brabender farinograph (Brabender GmbH & Co. KG, Duisburg, Germany). Each leavened dough (80 g) was placed in the mixing bowl and mixed for 5 min. Consistency was expressed in Brabender Units (BU) 2 min after mixing.

#### 2.3.9. Statistical Analysis

All results are expressed as means ± standard deviation of three replicates. The Tukey test and one-way ANOVA using Duncan’s multiple comparison test at 95% confidence level (*p* ≤ 0.05) were performed to evaluate sample differences. Samples showing *p* < 0.05 were considered significantly different.

## 3. Results

### 3.1. Effect of the Drying Process on Microbial Viability

The results of the drying process on the LAB population from the different treatments are shown in Table 1. Treatment T5 exhibited the lowest viability of LAB, followed by treatment BI2. This could be associated with the low stability of the microbiota at pH < 4.0 [15] and the effect of the drying process. On the other hand, once the sourdoughs were dried, the LAB population decreased significantly, as expected, due to the low water activity of the matrix [16]. However, after the reactivation of the sourdoughs, the LAB population partly recovered and after 24 h of fermentation, they reached the same population as before the drying process. This result agrees with the report by Santos et al. [17], in which they state that the drying of sourdoughs does not appear to affect the viability of sourdoughs’ microbiota.

For the total yeasts (Table 2), there appeared to be greater resistance to the drying process. This could be attributed to the high resistance of certain yeast species, especially those found in sourdough [18]. The yeast population was lower than that of LAB, as is typically expected in sourdoughs. On the other hand, when drying conditions are applied to sourdoughs, yeasts often exhibit more thermal resistance than LAB. AB addition did not appear to influence yeast population, meaning there were no residual yeasts from the tequila-making process that could interfere during the sourdough fermentation.

For total aerobic mesophilic bacteria, a pattern similar to that of LAB was observed (Table 3). This could be explained by the fact that some LAB species are also mesophilic [19]. However, after the drying process, the total mesophilic aerobic bacteria decreased considerably, and even after the reactivation process, the cell population remained lower than that of LAB, leading to the possibility that the drying of sourdough may be beneficial in reducing aerobic mesophilic bacteria in the products, which may be used as a method to control fermentation’s biochemical processes.

As expected, fungal growth was not detected (<10 CFU/g), since the doughs were all prepared under hygienic conditions to prevent fungal spoilage. Overall, the sourdough microbiota viability was influenced by the drying conditions and partial recovery was observed after the reactivation process (almost reaching the viability shown by the fresh sourdough after a 24 h fermentation), meaning that drying conditions do not affect the microbiota viability in a way that compromises the generation of bioactive compounds. Drying conditions can be used to easily manipulate a sourdough while preserving its beneficial properties.

### 3.2. Determination of Total Phenolic Compounds of the Sourdoughs

**Figure 1:**
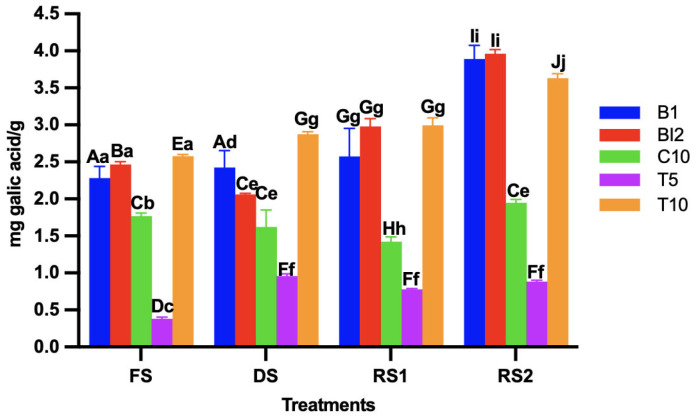
Total phenolic compounds content in the sourdoughs (B1: wheat sourdough; BI2: wheat sourdough inoculated with *L. lactis*; C10: agave bagasse with wheat flour; T5: wheat sourdough with 5% of agave added bagasse and inoculated with 1 × 10^6^ CFU/mL *L. lactis*; T10: wheat sourdough with 10% of agave bagasse added and inoculated with 1 × 10^6^ CFU/mL *L. lactis*) under different conditions (FS: fresh sourdough, DS: dried sourdough, RS1: reactivated sourdough at time 0, RS2: reactivated sourdough after 24 h). Different letters indicate a statistically significant difference (*p* < 0.05) in the same treatment (lower case) or between treatments (upper case).

While the total phenolic compound content decreased to a certain degree in the dried sourdoughs (Figure 1), it increased from 1% to 5% in all treatments once the sourdoughs were re-activated. However, no statistically significant differences were observed between the phenolic content in fresh and dried sourdough. Interestingly, the treatments T5 and C10 showed the lowest concentrations of total phenolic compounds in fresh and dried sourdough.

The reactivated treatments (RS1, RS2) showed higher levels of phenolic compounds than FS, regardless of the fermentation time. This could be due to the fermentation process and LAB metabolic activity in the sourdough, which is particularly associated with the production of free phenolic acids resulting from esterase and glycosidase activities [20]. This is corroborated by these results, as the treatments with the added strain of *Lactococcus lactis* (BI2, T10) exhibited higher phenolic levels than those without bacteria (C10). Following these results, it has been reported by Chulibert et al. [21] that phenolic content may be relatively stable under the thermal conditions, such as temperature, used in this study. AB may also act as a source of bioactive compounds.

### 3.3. Total Flavonoid Content of the Sourdoughs

**Figure 2:**
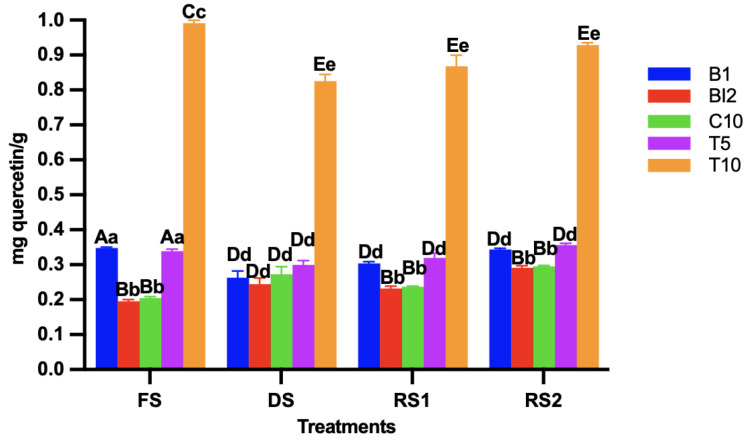
Total flavonoid content in the sourdoughs (B1: wheat sourdough; BI2: wheat sourdough inoculated with *L. lactis*; C10: agave bagasse with wheat flour; T5: wheat sourdough with 5% of agave bagasse added and inoculated with 1 × 10^6^ CFU/mL *L. lactis*; T10: wheat sourdough with 10% of agave bagasse added and inoculated with 1 × 10^6^ CFU/mL *L. lactis*) under different conditions (FS: fresh sourdough, DS: dried sourdough, RS1: reactivated sourdough at time 0, RS2: reactivated sourdough after 24 h). Different letters indicate a statistically significant difference (*p* < 0.05) in the same treatment (lower case) or between treatments (upper case).

Sourdough fermentation is known to enhance the release of bioactive compounds due to the combined activity of both lactic acid bacteria and yeasts [22]. Among the treatments, T10 exhibited the highest flavonoid content (expressed as meq catechin/g of sourdough), followed by B1 (*p* < 0.05) (Figure 2). These results could be attributed to the influence of *L. lactis* in releasing flavonoids from the AB, thereby leading to an increase in bioactive compounds [23]. On the other hand, the drying method appeared to have a greater impact on flavonoids than on phenolic content, likely due to the effects of both temperature and processing time [24]. Although flavonoid content partly increased after the 24 h fermentation following reactivation, their levels remained lower than those observed in freshly made sourdough, indicating that the drying process directly affected their availability.

Interestingly, all treatments showed higher levels of flavonoids in T10, no matter the drying conditions, suggesting that the ingredients may have a greater impact on the bioactive compounds content than the process conditions.

### 3.4. Determination of the Antioxidant Capacity by DPPH and ABTS Method

**Figure 3:**
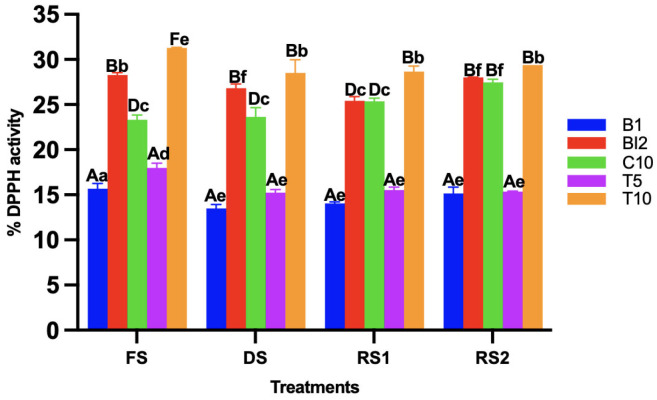
DPPH activity of the different sourdoughs (B1: wheat sourdough; BI2: wheat sourdough inoculated with *L. lactis*; C10: agave bagasse with wheat flour; T5: wheat sourdough with 5% of agave bagasse added and inoculated with 1 × 10^6^ CFU/mL *L. lactis*; T10: wheat sourdough with 10% of agave bagasse added and inoculated with 1 × 10^6^ CFU/mL *L. lactis*) under different conditions (FS: fresh sourdough, DS: dried sourdough, RS1: reactivated sourdough at time 0, RS2: reactivated sourdough after 24 h). Different letters indicate a statistically significant difference (*p* < 0.05) in the same treatment (lower case) or between treatments (upper case).

**Figure 4:**
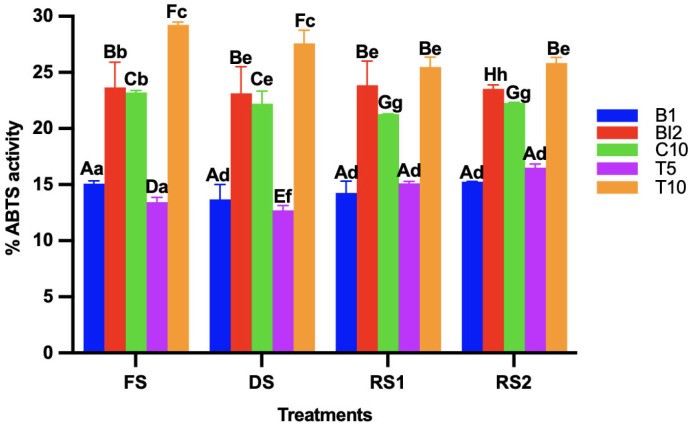
ABTS activity of the different sourdoughs (B1: wheat sourdough; BI2: wheat sourdough inoculated with *L. lactis*; C10: agave bagasse with wheat flour; T5: wheat sourdough with 5% of agave bagasse added and inoculated with 1 × 10^6^ CFU/mL *L. lactis*; T10: wheat sourdough with 10% of agave bagasse added and inoculated with 1 × 10^6^ CFU/mL *L. lactis*) under different conditions (FS: fresh sourdough, DS: dried sourdough, RS1: reactivated sourdough at time 0, RS2: reactivated sourdough after 24 h). Different letters indicate a statistically significant difference (*p* < 0.05) in the same treatment (lower case) or between treatments (upper case).

During sourdough fermentation, antioxidant capacity increases mainly due to the release of bound phenolic compounds and the enhanced solubility of polyphenols. For both the DPPH (Figure 3) and ABTS methods (Figure 4), the treatments with the highest antioxidant activity were those in which AB was added at a 10% concentration. This could be attributed to the increased bioactive compounds introduced by the agave, as it has been reported that sourdoughs made with high-fiber ingredients are more likely to enhance their antioxidant capacity. This is due to the presence of cellulase enzymes in sourdough, which may promote the release of phenolic compounds [25].

In agreement with previous results, antioxidant activity is more related to the ingredients and their ratio than to the processing conditions.

The drying process reduced the antioxidant capacity in all treatments, as expected, since antioxidant capacity can decrease due to factors such as temperature. However, once the sourdoughs were re-activated, the antioxidant capacity reached higher levels than those observed before the drying process. This suggests that several bioactive compounds may have been released during drying, and their availability may have increased during fermentation [26]. Overall, the antioxidant capacity increased for the treatments where AB was added, indicating that fiber can act as a source of phenolic and flavonoid compounds. Additionally, lipolytic enzymes likely secreted by LAB may have contributed to the degradation of these compounds, leading to an increase in antioxidant capacity [27].

### 3.5. Determination of Free Amino Acid Content (OPA)

**Figure 5:**
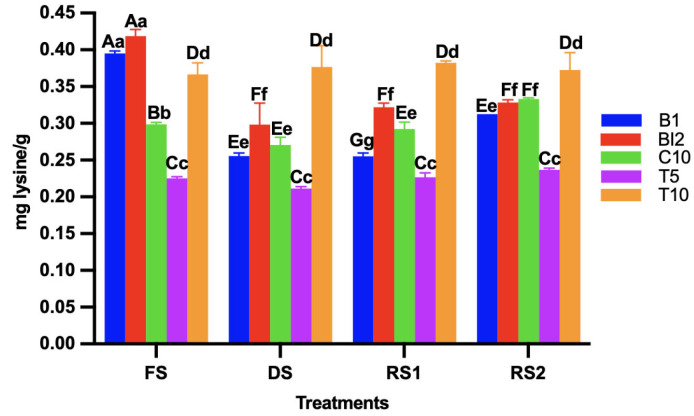
Free peptides and amino acid content in the different sourdoughs (B1: wheat sourdough; BI2: wheat sourdough inoculated with *L. lactis*; C10: agave bagasse with wheat flour; T5: wheat sourdough with 5% of agave bagasse added and inoculated with 1 × 10^6^ CFU/mL *L. lactis*; T10: wheat sourdough with 10% of agave bagasse added and inoculated with 1 × 10^6^ CFU/mL *L. lactis*) under different conditions (FS: fresh sourdough, DS: dried sourdough, RS1: reactivated sourdough at time 0, RS2: reactivated sourdough after 24 h). Different letters indicate a statistically significant difference (*p* < 0.05) in the same treatment (lower case), or between treatments (upper case).

Proteolytic activity is strongly correlated with the fermentation capacity of sourdough microbiota, but it often depends on the strain and incubation process of the sourdough [28]. To assess the degree of proteolysis (determined by the amount of free amine groups in the sample), the OPA method was used. In all sourdoughs, an increase in free amine groups and peptides was observed during fermentation, with no significant difference in concentration when the sourdough was dried (Figure 5). Higher levels of amine groups and peptides were found when the external strain was added, consistent with the findings of Khanlari et al. [29], who reported that inoculating sourdoughs with co-cultures (multiple strains) enhances proteolytic activity, leading to an increase in free amine groups and peptide content.

### 3.6. Volume and Consistency of the Dough

**Figure 6:**
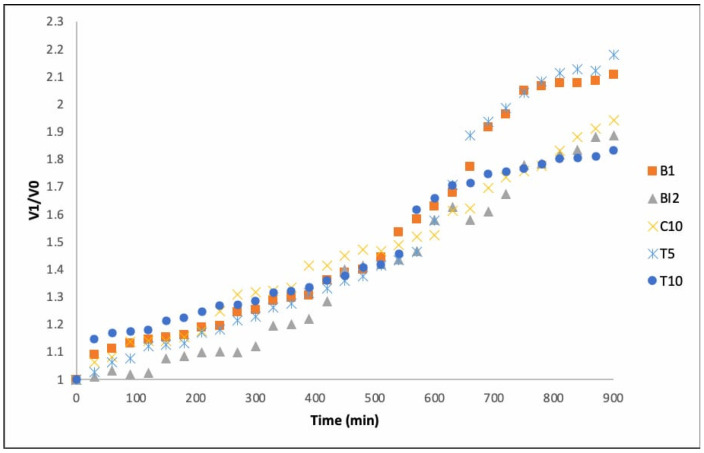
Dough volumes during leavening time utilizing different samples. B1: wheat sourdough; BI2: wheat sourdough inoculated with *L. lactis*; C10: agave bagasse with wheat flour; T5: wheat sourdough with 5% of agave bagasse added and inoculated with 1 × 10^6^ CFU/mL *L. lactis*; T10: wheat sourdough with 10% of agave bagasse added and inoculated with 1 × 10^6^ CFU/mL *L. lactis*.

When sourdough is used in bread formulation, it must be incorporated with more flour so that the consistency of the final bread is reached. In this sense, it is important to monitor the volume of the roll doughs, since volume is often an indicator of the carbon dioxide generated, mostly from the yeast present in the sourdough [12]. On the other hand, when formulating new bakery products with added fiber sources, such as AB, consistency is often a critical parameter compromising bread acceptance because of density reduction [30]. Figure 6 shows the volumetric development of the doughs over 900 min of fermentation, expressed as the V_1_/V_0_ ratio (final volume relative to initial volume). The objective was to evaluate how the addition of agave bagasse (AB), either alone or in combination with *Lactococcus lactis*, influences the leavening ability of the dough—an indirect indicator of microbial activity and CO_2_ production during fermentation.

Among the five treatments, T5 (5% AB + *L. lactis*) showed the greatest volume increase, reaching a V_1_/V_0_ ratio of approximately 2.2 at the end of fermentation. T10 (10% AB + *L. lactis*) followed a similar trend, although with a slightly lower final volume. This suggests a synergistic effect between AB and *L. lactis*, where the presence of fermentable fibers and the metabolic activity of lactic acid bacteria jointly enhance gas production, resulting in greater dough expansion.

The control sourdough (B1) also showed good leavening performance, confirming the effectiveness of traditional fermentation. However, both C10 (wheat flour + AB, without inoculum) and BI2 (sourdough + *L. lactis* only) showed lower volume increases (with final V_1_/V_0_ ratios of approximately ~1.8 and ~1.7, respectively). These results indicate that neither AB nor *L. lactis* alone is sufficient to enhance fermentation as effectively as their combination.

On the other hand, the consistency of the doughs, assessed as when the dough had reached a suitable time for baking, showed different values among the samples (Table 4), with significant differences in most treatments. The treatments where AB was added showed lower consistency (from 408 to 496) than those without AB (from 456 to 488), meaning that the addition of AB had a significant impact on the dough’s technological properties, as expected, since AB is mostly added as a fiber source, meaning that, while the dough volume did not appear to be affected by the AB addition, it affected the consistency of the sourdoughs.

The reactivation time impacted the sourdough consistency, likely due to the slow reactivation, which could favor the production of exopolysaccharides from LAB and CO_2_ from the yeasts. The highest fermentation volumes were observed for doughs containing 10% AB and the added *L. lactis* strain, whereas consistency values were low when only AB was added. For dried sourdough, the values were lower than in all other treatments, as expected, since dried sourdough is in the form of a powder and, therefore, does not give the consistency that fresh or re-activated sourdough provides.

## 4. Conclusions

The growing popularity of sourdough in the market has driven the need for innovative ingredients that enhance its nutritional and functional properties. This study demonstrated that the addition of agave bagasse (AB) and *Lactococcus lactis* can be effective strategies to improve sourdough while maintaining its functional and technological qualities. The inclusion of AB increased the content of phenolic compounds and flavonoids, enhancing the antioxidant capacity of the sourdough. Similarly, *Lactococcus lactis* contributed to the release of bioactive compounds through its enhanced fermentation activity, further enriching the nutritional profile.

During the fermentation process, proteolytic activity increased, suggesting that the microbiota present in the sourdough effectively utilized the fiber from agave bagasse. This indicates that AB can serve as a valuable functional ingredient, contributing to the overall nutritional value without compromising the consistency and volume of the dough during leavening.

The study also highlighted that the drying process at lower temperatures had a minimal impact on the sourdough’s bioactive compounds and antioxidant capacity. Although drying initially reduced microbial viability, the reactivation process effectively restored these properties, confirming the stability and resilience of the sourdough microbiota. This finding suggests that drying and reactivation can be safe and efficient methods for preserving and transporting sourdough, potentially reducing production and logistics costs.

Overall, this study underscores the potential of using agave bagasse as a sustainable ingredient in sourdough production, enhancing its nutritional and functional attributes while promoting eco-friendly bakery products. The combined use of AB and *L. lactis* could provide a novel approach for developing value-added baked goods, aligning with consumer demand for healthier and more sustainable food choices.

## Figures and Tables

**Table 1:** Microbial counts of lactic acid bacteria of samples (B1: wheat sourdough; BI2: wheat sourdough inoculated with *L. lactis*; C10: agave bagasse with wheat flour; T5: wheat sourdough with 5% of agave bagasse added and inoculated with 1 × 10^6^ CFU/mL *L*. *lactis*; T10: wheat sourdough with 10% of agave bagasse added and inoculated with 1 × 10^6^ CFU/mL *L. lactis*) under different processing conditions (FS: fresh mature sourdough; DS: dried sourdough; RS1: dried sourdough re-activated; RS2: reactivated sourdough after 24 h).

Treatment		Lactic Acid Bacteria Count (log CFU/g)			
	pH	FS	DS	RS1	RS2
B1	4.56 ± 0.12 ^a^	8.3 ± 0.04 ^aA^	6.9 ± 0.13 ^aB^	7.2 ± 0.45 ^aB^	7.9 ± 0.34 ^aC^
BI2	3.89 ± 0.34 ^b^	8.8 ± 0.63 ^bD^	5.9 ± 0.05 ^bE^	6.9 ± 0.67 ^bF^	7.4 ± 0.45 ^bG^
C10	4.89 ± 0.66 ^c^	8.9 ± 0.45 ^bH^	5.3 ± 0.98 ^cI^	6.1 ± 0.34 ^cI^	8.3 ± 0.67 ^cJ^
T5	4.30 ± 0.04 ^d^	8.3 ± 0.56 ^aK^	6.9 ± 0.06 ^aL^	6.3 ± 0.45 ^cM^	7.5 ± 0.06 ^dN^
T10	4.63 ± 0.56 ^a^	8.9 ± 0.23 ^bO^	6.8 ± 0.56 ^aP^	7.4 ± 0.98 ^dQ^	8.6 ± 0.56 ^eO^

Different letters indicate significant differences (*p* < 0.05) per Tukey’s test.

**Table 2:** Microbial counts of total yeast of samples (B1: wheat sourdough; BI2: wheat sourdough inoculated with *L. lactis*; C10: agave bagasse with wheat flour; T5: wheat sourdough with 5% of agave bagasse added and inoculated with 1 × 10^6^ CFU/mL *L. lactis*; T10: wheat sourdough with 10% of agave bagasse added and inoculated with 1 × 10^6^ CFU/mL *L. lactis*) under different processing conditions (FS: fresh mature sourdough; DS: dried sourdough; RS1: dried sourdough re-activated; RS2: reactivated sourdough after 24 h).

Treatment		Yeast Count (log CFU/g)			
	pH	FS	DS	RS1	RS2
B1	4.56 ± 0.12 ^a^	5.9 ± 0.04 ^aA^	5.3 ± 0.13 ^aB^	5.2 ± 0.45 ^aC^	5.9 ± 0.34 ^aA^
BI2	3.89 ± 0.34 ^b^	5.2 ± 0.63 ^bD^	5.0 ± 0.05 ^bE^	5.9 ± 0.67 ^bF^	5.6 ± 0.45 ^bF^
C10	4.89 ± 0.66 ^c^	5.7 ± 0.45 ^aG^	5.5 ± 0.98 ^cH^	5.3 ± 0.34 ^aI^	5.3 ± 0.67 ^cI^
T5	4.30 ± 0.04 ^d^	5.3 ± 0.56 ^bJ^	4.5 ± 0.06 ^dK^	5.8 ± 0.45 ^bL^	5.5 ± 0.06 ^dJ^
T10	4.63 ± 0.56 ^a^	5.9 ± 0.23 ^aK^	5.0 ± 0.56 ^bL^	5.4 ± 0.98 ^aM^	5.6 ± 0.56 ^eN^

Different letters indicate significant differences (*p* < 0.05) per Tukey’s test.

**Table 3:** Microbial counts of total aerobic mesophilic bacteria of samples (B1: wheat sourdough; BI2: wheat sourdough inoculated with *L. lactis*; C10: agave bagasse with wheat flour; T5: wheat sourdough with 5% of agave bagasse added and inoculated with 1 × 10^6^ CFU/mL *L. lactis*; T10: wheat sourdough with 10% of agave bagasse added and inoculated with 1 × 10^6^ CFU/mL *L. lactis*) under different processing conditions (FS: fresh mature sourdough; DS: dried sourdough; RS1: dried sourdough re-activated; RS2: reactivated sourdough after 24 h).

Treatment		Mesophilic Bacteria Count (log CFU/g)			
	pH	FS	DS	RS1	RS2
B1	4.56 ± 0.12 ^a^	7.9 ± 1.02 ^aA^	6.3 ± 0.13 ^aB^	7.1 ± 0.45 ^aC^	7.1 ± 0.34 ^aC^
BI2	3.89 ± 0.34 ^b^	7.2 ± 0.98 ^bD^	6.0 ± 0.05 ^bE^	6.9 ± 0.67 ^aD^	7.0 ± 0.45 ^aD^
C10	4.89 ± 0.66 ^c^	7.3 ± 0.05 ^bF^	6.5 ± 0.98 ^cG^	6.5 ± 0.34 ^bG^	6.9 ± 0.67 ^aH^
T5	4.30 ± 0.04 ^d^	7.6 ± 0.36 ^cI^	6.1 ± 0.06 ^bJ^	7.1 ± 0.45 ^aK^	7.2 ± 0.06 ^aM^
T10	4.63 ± 0.56 ^a^	7.2 ± 0.13 ^bN^	6.6 ± 0.56 ^dO^	6.9 ± 0.98 ^aP^	7.0 ± 0.56 ^aP^

Different letters indicate significant differences (*p* < 0.05) per Tukey’s test.

**Table 4:** Consistency of the sourdoughs (B1: wheat sourdough; BI2: wheat sourdough inoculated with *L. lactis*; C10: agave bagasse with wheat flour; T5: wheat sourdough with 5% of agave bagasse added and inoculated with 1 × 10^6^ CFU/mL *L. lactis*; T10: wheat sourdough with 10% of agave bagasse added and inoculated with 1 × 10^6^ CFU/mL *L. lactis*) under different processing conditions (FS: fresh sourdough, DS: dried sourdough, RS1: reactivated sourdough at time 0, RS2: reactivated sourdough after 24 h).

	Consistency (BU)				
Treatment	B1	B2	C10	T5	T10
FS	488.45 ± 1.34 ^aA^	489.34 ± 0.35 ^aA^	488.23 ± 1.63 ^aA^	477.45 ± 0.94 ^aB^	498.34 ± 1.45 ^cC^
DS	390.21 ± 0.23 ^fF^	394.32 ± 0.34 ^fF^	387.34 ± 0.83 ^fF^	391.23 ± 0.56 ^fF^	38723 ± 0.34 ^fF^
RS1	448.33 ± 1.97 ^bB^	433.66 ± 0.98 ^cD^	392.33 ± 1.34 ^dE^	409.66 ± 1.23 ^dE^	458.66 ± 0.87 ^bB^
RS2	456.34 ± 0.34 ^cC^	443.34 ± 1.24 ^bC^	476.34 ± 1.98 ^aB^	410.34 ± 0.34 ^dE^	408.45 ± 1.34 ^dE^

Different letters indicate a statistically significant difference (*p* < 0.05) in the same treatment (lower case) or between treatments (upper case).

## Data Availability

The original contributions presented in the study are included in the article, further inquiries can be directed to the corresponding author.
